# Supplementary material for: Unraveling the Molecular Mechanisms of Tomatoes’ Defense against Botrytis cinerea: Insights from Transcriptome Analysis of Micro-Tom and Regular Tomato Varieties
Source: Plants (Basel). 2023 Aug 16;12(16):2965. doi: 10.3390/plants12162965 (PMC10459989; doi:10.3390/plants12162965)
Supplement: Supplementary file 1 [file plants-12-02965-s001.zip › plants-2527061-supplementary/Figure S1-S4.pdf]

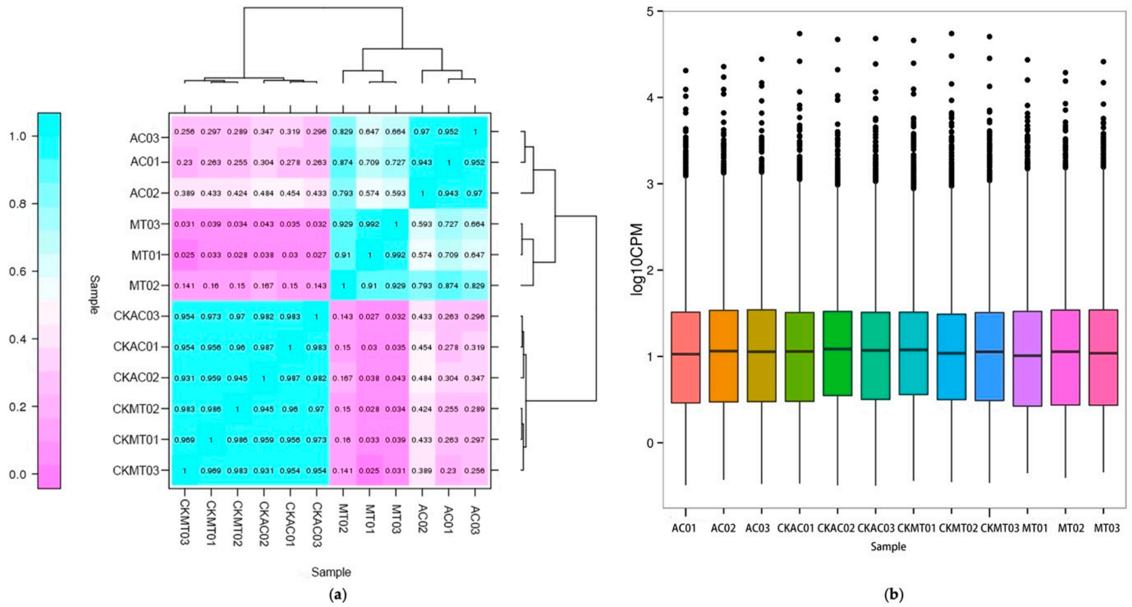

Figure S1. Correlation Heatmap of Gene Expression Levels between Sample Pairs and Box plots of CPM values for each sample. (a) Biological replicate correlation testing and assessment of the reliability of differentially expressed genes and screening of outlier samples. The Pearson correlation coefficient (r) is used as an evaluation metric for the biological replicate correlation; (b) Box plot of gene expression distribution of all samples. The abscissa in the figure represents different samples, and the ordinate represents the logarithmic value of the sample expression CPM. No *B. cinerea* at CKAC01, CKAC02, CKAC03 and CKMT01, CKMT02, CKMT03; *B. cinerea* treatment at AC01, AC02, AC03 and MT01, MT02, MT03.

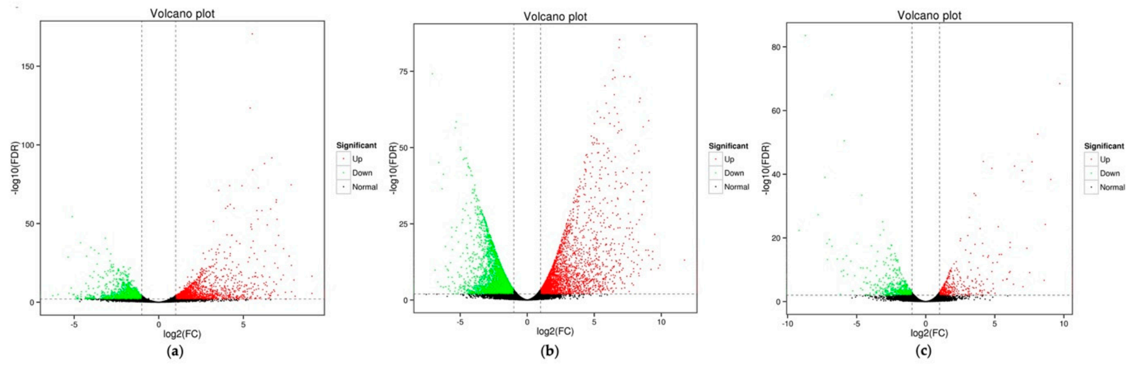

Figure S2. Differentially expressed genes. (a) CKAC _vs_ AC; (b) CKMT _vs_ MT; (c) AC _vs_ MT. Volcano Plot displays the differences in gene expression levels between two samples or groups, along with their statistical significance. Each point represents a gene, where the x-axis represents the log-fold change in gene expression between the two samples, and the y-axis represents the negative logarithm of the statistical significance of the gene expression change. Green points represent downregulated differentially expressed genes, red points represent upregulated differentially expressed genes, and black points represent non-differentially expressed genes.

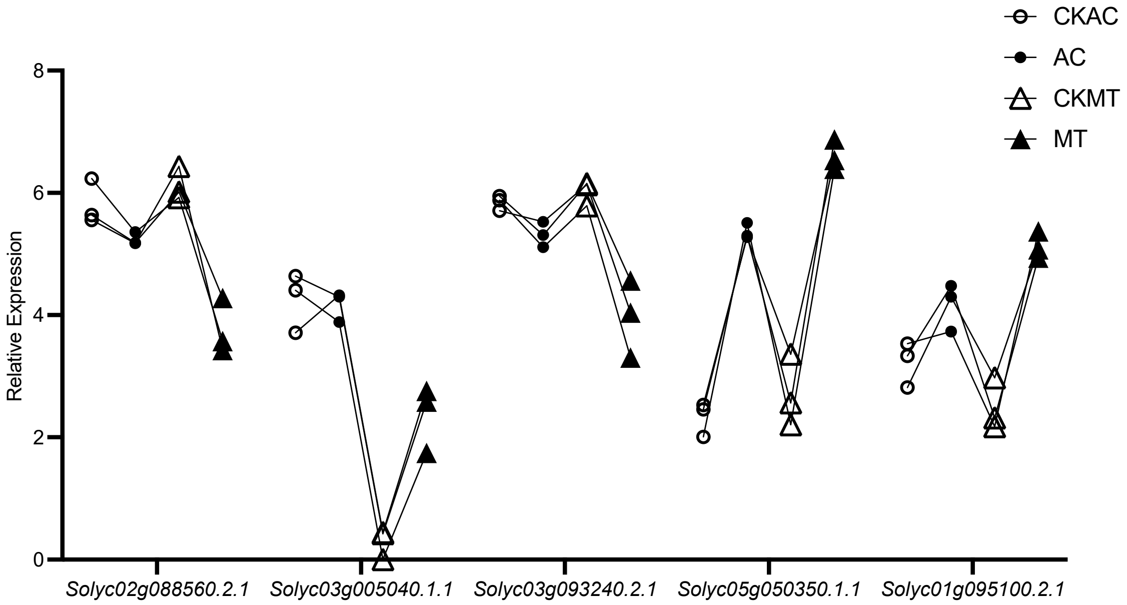

Figure S3. A comprehensive depiction of the expression levels of differentially expressed KEGG analysis plant-pathogen interaction related 5 genes among the four groups (CKAC, AC, CKMT, MT) in leaves at 22 h (AC) and 24 h (MT) post-inoculation with *B. cinerea*.

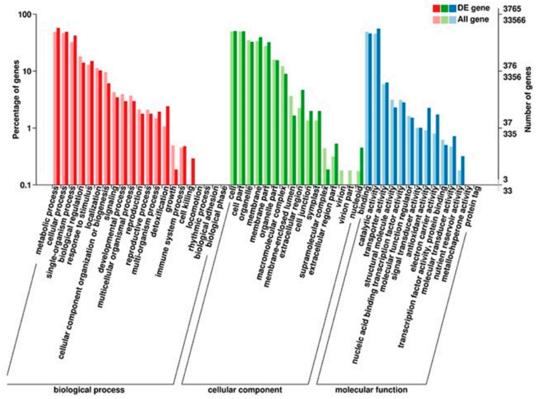

(a)

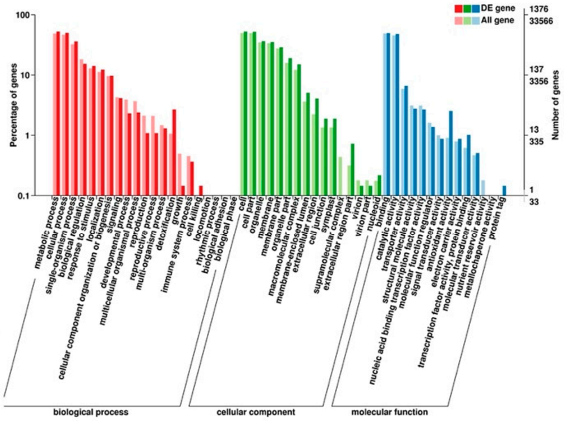

(c)

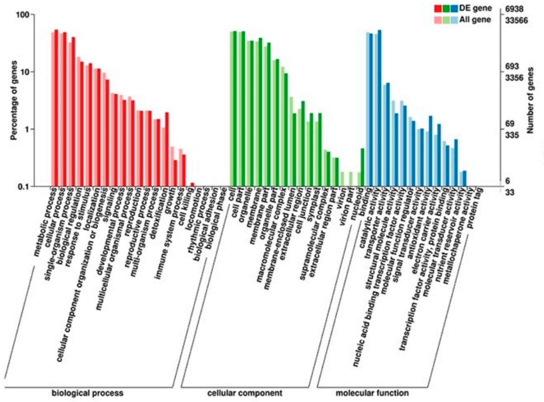

(b)

Figure S4. The GO enrichment analysis of DEGs. (a) CKAC _vs_ AC; (b) CKMT _vs_ MT；(c) AC _vs_ MT. The x-axis represents the GO terms, while the y-axis on the left indicates the percentage of genes and on the right indicates the number of genes.
